# Extreme ultraviolet microscope characterization using photomask surface roughness

**DOI:** 10.1038/s41598-020-68588-w

**Published:** 2020-07-15

**Authors:** Gautam Gunjala, Antoine Wojdyla, Stuart Sherwin, Aamod Shanker, Markus P. Benk, Kenneth A. Goldberg, Patrick P. Naulleau, Laura Waller

**Affiliations:** 1 Department of Electrical Engineering and Computer Sciences, University of California, Berkeley, Berkeley, CA 94720 USA; 2 Advanced Light Source, Lawrence Berkeley National Lab, Berkeley, CA 94720 USA; 3 Center for X-Ray Optics, Lawrence Berkeley National Lab, Berkeley, CA 94720 USA

**Keywords:** Computational science, Optical techniques

## Abstract

We demonstrate a method for characterizing the field-dependent aberrations of a full-field synchrotron-based extreme ultraviolet microscope. The statistical uniformity of the inherent, atomic-scale roughness of readily-available photomask blanks enables a self-calibrating computational procedure using images acquired under standard operation. We characterize the aberrations across a 30-um field-of-view, demonstrating a minimum aberration magnitude of smaller than $\lambda /21 \, {\hbox {rms}}$ averaged over the center 5-um area, with a measurement accuracy better than $\lambda /180 \, {\hbox {rms}}$. The measured field variation of aberrations is consistent with system geometry and agrees with prior characterizations of the same system. In certain cases, it may be possible to additionally recover the illumination wavefront from the same images. Our method is general and is easily applied to coherent imaging systems with steerable illumination without requiring invasive hardware or custom test objects; hence, it provides substantial benefits when characterizing microscopes and high-resolution imaging systems in situ.

## Introduction

Measuring and correcting the aberrations of full-field imaging systems is a widely understood problem for which many methods have been developed. However, these methods are generally difficult, costly, or impractical to apply to specialized modalities such as extreme ultraviolet (EUV) imaging. Interferometric techniques^1–7^ or adaptive optics, while widely used in the visible spectrum^8–11^, require complicated and expensive hardware, making them impractical in the EUV regime. One measurement approach used in EUV is to image precisely-calibrated test objects^12,13^ such as gratings^14,15^, contact arrays^16,17^, or custom features^18^, which do not require modifying the system hardware. However, known test objects containing features with sizes near the resolution limit of the imaging system are difficult or expensive to fabricate with high fidelity. Furthermore, many of these test objects are not ideal for aberration and wavefront recovery; for example, periodic targets only probe discrete points in frequency space, requiring many feature sizes and orientations to be measured through focus^14^. In addition, fabrication errors and the 3D structure of test objects can complicate analysis^19,20^.

Here, we employ a method for characterizing the aberrations of a full-field imaging system that does not require hardware modifications or the fabrication of test objects. We apply the method to the SHARP High-NA Actinic Reticle Review Project (SHARP)^21^, an EUV microscope that operates near 13.5 nm wavelength, but we emphasize that it is suitable for any full-field imaging system that has coherent, steerable illumination. To implement, we acquire speckle images of a suitable object at multiple angles of plane-wave illumination. The object being imaged does not need to be precisely fabricated; it only needs to have a pseudo-random surface, weak phase and sufficient power-spectral density extending to the imaging system’s resolution limit. A blank EUV photomask conveniently meets these requirements, due to intrinsic surface roughness^22,23^. Similar methods have been demonstrated in optical microscopy, using a diffuser with index-matching oil^24,25^, and in electron microscopy, using amorphous carbon (i.e the Zemlin tableau method)^26–29^.

Our method has several advantages over other approaches that entail imaging test objects. First, no special fabrication is required, as suitable objects can be found opportunistically. Second, unlike periodic objects, uncorrelated surface roughness provides isotropic sampling of frequency space. Third, no registration or alignment of the test object is required, as the statistics of the roughness should not change across the object. Finally, our method is data-efficient; in our implementation, we use 10 speckle images to recover all field-varying aberrations of up to order 5.

## Experimental setup

**Figure 1:**
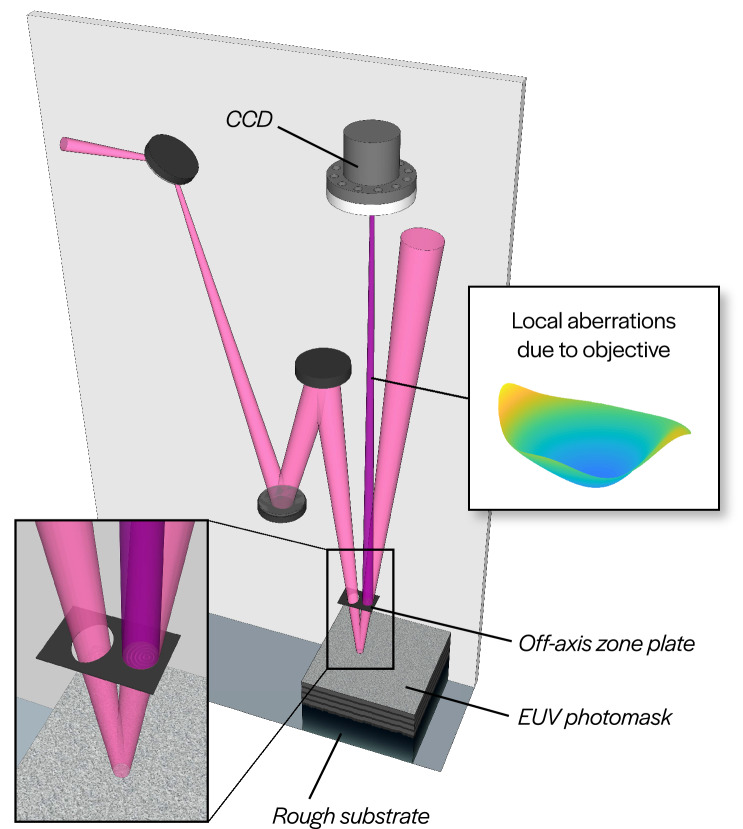
SHARP EUV microscope imaging configuration. A mirror conjugated with the object plane (which contains a blank EUV photomask) allows control over illumination angle. The objective lens (an off-axis Fresnel zone plate) images the beam scattered by the mask blank onto the sensor. The system suffers from field-dependent aberrations, primarily due to Petzval curvature. See “Methods” for additional details.

Experiments were performed on the SHARP microscope at Lawrence Berkeley National Laboratory’s Advanced Light Source (ALS). SHARP is a synchrotron-based, full-field EUV microscope designed to emulate aerial image formation in industrial EUV photolithography scanners. SHARP uses an angle-scanning mirror optically conjugated with the object plane^21,30^ for steerable illumination angles. A blank EUV photomask was coherently illuminated with a central ray angle of $6^\circ$ and imaged onto a CCD sensor using an off-axis Fresnel zoneplate lens, as shown in Fig. 1.

**Figure 2:**
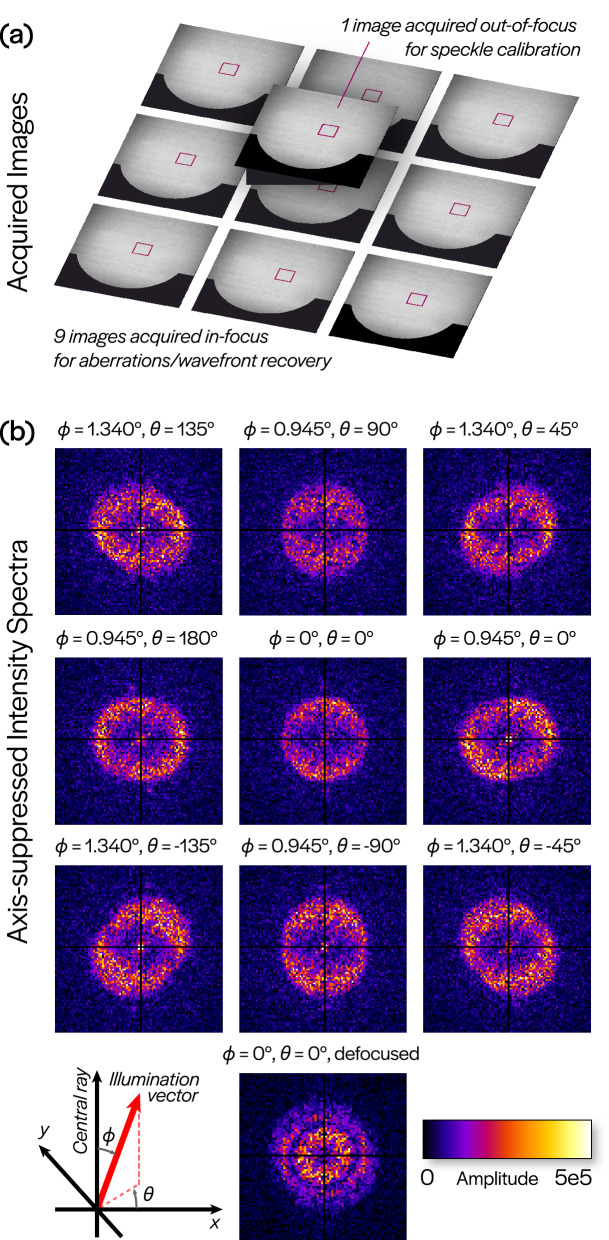
Measurements and computed Fourier spectra. (**a**) Ten intensity images (9 with varying illumination angle, 1 with defocus) are acquired by SHARP with a blank EUV photomask as the object. (**b**) Spatial spectra computed at the sub-region of the full field indicated by the magenta square in (**a**). Illumination angles are given with respect to the $6^\circ$ central ray angle, as shown by the schematic in the lower left. Note that these angles should be treated as inputs in the acquisition process, and do not account for angle variations across the FOV due to wavefront curvature.

The configuration of SHARP characterized in this paper features a zoneplate lens with an NA of 0.0825, a field-of-view (FOV) of approximately $30\times 30~\upmu \, {\hbox {m}}^2$ and an effective pixel size of 15 nm due to a magnification of 900$\times$ (see “Methods” for more“ details). For our analysis, we collected 10 coherently illuminated images of the photomask blank (Fig. 2). An image taken with central illumination and a large defocus was used to estimate speckle properties. The other 9 images were acquired with varying illumination angles near the central ray angle. The choice of these angles is discussed in the following section.

## Objective aberration characterization

Our technique is based on the Fourier optical model of coherent imaging systems, in which a complex-valued linear transfer function acts on an incident electric field, and the output is an intensity measurement. Mathematically, this can be written in terms of 2D spatial coordinate, ${\mathbf {x}}$, and 2D spatial frequency coordinate, ${\mathbf {u}}$ (normalized by ${\text {NA}}_{{\text {obj}}}/\lambda$), as

1 $$\begin{aligned} I_{{\text {out}}}\left( {\mathbf {x}} \right) = \left| {\mathscr {F}}^{-1} \left[ {\widehat{P}}\left( {\mathbf {u}} \right) \cdot {\mathscr {F}} \left[ E_{{\text {incident}}} \left( {\mathbf {x}} \right) \right] \right] \right| ^{2}, \end{aligned}$$

where ${\mathscr {F}}[\cdot ]$ denotes the Fourier transform. In Eq. (1), the transfer function of the imaging system, ${\widehat{P}}$, has the following structure:

2 $$\begin{aligned} {\widehat{P}}\left( {\mathbf {u}} \right) = \exp \left\{ i \cdot W \left( {\mathbf {u}} \right) \right\} \cdot {\text {circ}} \left( {\mathbf {u}} \right) , \end{aligned}$$

with a bandlimit set by the NA and the wavelength^31^. We refer to the phase of this transfer function, *W*, as the wavefront error function (WEF)—a real-valued function, typically expressed in the Zernike basis where coefficients map to canonical, space-invariant aberrations. These aberrations include defocus, astigmatism and coma, which arise from common alignment errors and aberrations inherent to the lens in use. We address field-varying aberrations by applying Eq. (1) locally to different segments of the full FOV.

Within this framework, the structure of blank photomasks allows the derivation of a simplified imaging forward model relating illumination angle and aberrations—both inherently wavefront characteristics—to *intensity* images. Blank EUV photomasks have an intrinsic random surface roughness on the order of 0.2 nm^32^ and can be modeled as stationary random weak phase objects^33–35^. Under coherent illumination, they generate speckle with dense and wide angular spectrum that acts as a probe of the system’s transfer function. Due to stationarity, the photomask surface can be adequately described by a few statistical parameters; we need not know its precise surface shape. These properties enable the use of a forward model that describes the spatial Fourier spectrum of an intensity measurement, $\widehat{I_{\varnothing ,j}}$, under plane wave illumination angles indexed by *j*, and computational DC-suppression. The model, derived in^25^, is:

3 $$\begin{aligned} \widehat{I_{\varnothing ,j}}\left( {\mathbf {u}} \right) = i \cdot \left[ \eta \left( {\mathbf {u}} \right) \circ \widehat{\varphi _d} \left( {\mathbf {u}} \right) \right] \circ \left[ \widehat{P^{*}}\left( {\mathbf {u}}_j \right) {\widehat{P}}\left( \mathbf {u+u}_j \right) - {\widehat{P}}\left( {\mathbf {u}}_j \right) \widehat{P^{*}}\left( \mathbf {-u+u}_j \right) \right] , \end{aligned}$$

where $\eta \left( {\mathbf {u}} \right) \sim {\text {Rayleigh}}\left( \xi \right)$ are independent and identically distributed, $\widehat{{\varphi }_d}({\mathbf {u}})$ is a deterministic Gaussian support function related to the mean surface roughness, and $\circ$ denotes element-wise multiplication. The Rayleigh distribution parameter, $\xi$, is also related to the surface roughness and can be estimated from data (see “Methods”).

**Figure 3:**
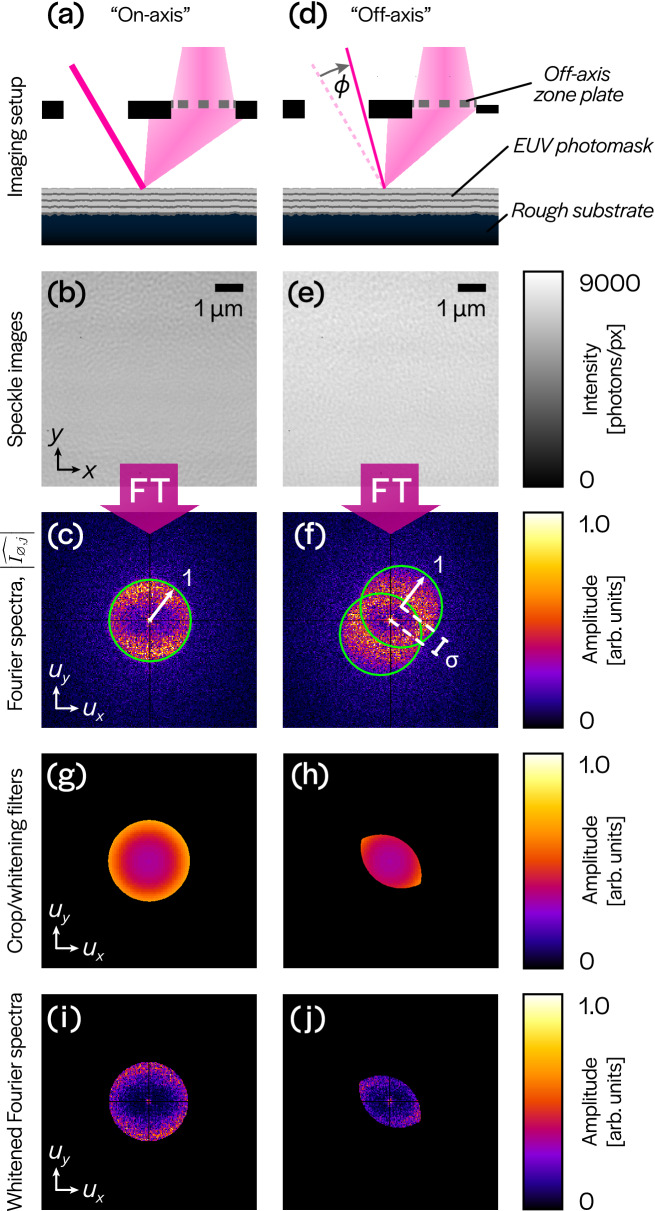
Coherent speckle imaging under on- and off-axis illumination. (**a**,**b**) Illumination with a $6^\circ$ central ray angle produces an effective on-axis speckle image of the photomask. (**c**) Fourier spectrum magnitude of on-axis measurement. (**d**,**e**) Illumination with deviation of $\phi$ from the $6^\circ$ central ray angle produces an off-axis image of the photomask. (**f**) Fourier spectrum magnitude demonstrating a shift of $\sigma = \sin \phi / {\text {NA}}_{{\text {obj}}}$. (**g**,**h**) Filters that crop the spectral magnitudes to their interference regions and divide out the Gaussian window $|{\widehat{\varphi }}_d|$ to whiten the residual noise. (**i**,**j**) Whitened Fourier spectra computed by applying filters (**g**,**h**) to spectra (**c**,**f**), respectively.

From this equation, we can see that the pupil function ${\widehat{P}}$ and its conjugate each result in a circular support region. By changing the angle of illumination, ${\mathbf {u}}_j$, the circular regions translate polar-symmetrically, thus changing the overlap region and the interference pattern within. The change in overlap region can be seen in the supports of Fig. 3g,h. For aberration recovery, the region-of-interest is where the two circular supports overlap and produce interference patterns (e.g. Fig. 3c,f). A set of blank-photomask intensity images acquired with different illumination angles will uniquely identify the phase in the pupil (the WEF), and hence the aberrations, as long as the chosen angles provide sufficient diversity of interference patterns. For best results, illumination angles should be chosen such that the deflection angle, $\phi$, satisfies $0.2< \sin \phi / {\text {NA}}_{{\text {obj}}} < 0.3$. This ensures that the resulting interference patterns are distinct from those produced by on-axis illumination, and that they exist within a sufficiently large overlap region. Using a variety of azimuthal angles, $\theta$, further diversifies the interference patterns in measurements. We model the spatial Fourier spectrum of the photomask as an instance of white noise, $\eta$, within a Gaussian support, $|\widehat{\varphi _d}|$^24,25^. Since the atomic-scale features on the photomask are smaller than the imaging resolution, this Gaussian support extends beyond the imaging bandlimit and ensures that the object probes the entire pupil.

**Figure 4:**
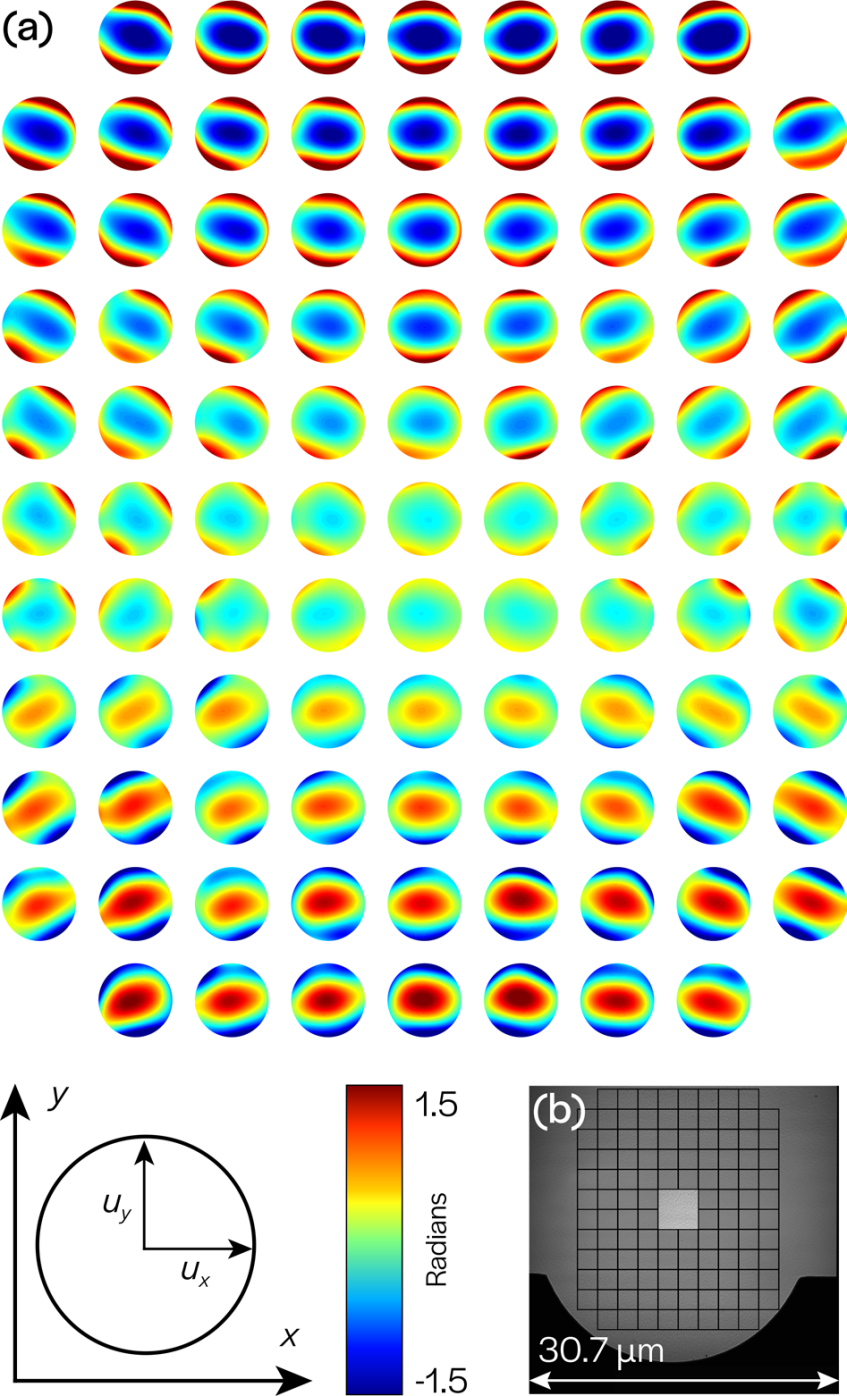
Field-dependent aberrations. (**a**) Wavefront error functions (WEFs) plotted across the field-of-view (FOV), demonstrating minimal aberrations in the center and an increase in magnitude at edges. Each WEF is a function of $(u_x,u_y)$ within the unit disk and corresponds to an (*x*, *y*) position in the FOV, as shown in the schematic in the lower left. (**b**) Square sub-regions of the full FOV show the (*x*, *y*) positions represented by the WEFs, with one sub-region highlighted to demonstrate size.

To apply our technique to the SHARP microscope, we segmented the full $2048 \times 2048$ pixel FOV into $256 \times 256$ pixel sub-regions with 50% overlap in the horizontal and vertical directions. Sub-regions in which the photomask was occluded or did not provide sufficient contrast (near the boundaries) were excluded. The defocused image was used to estimate the Gaussian support, $\widehat{\varphi _d}$, and distribution parameter, $\xi$. For each sub-region, the other 9 images were cropped to the sub-region boundary and the magnitudes of their Fourier spectra were computed. Pixel values in a small neighborhood around the DC frequency were set to zero in each spectrum. Noise-whitening filters were then applied to the spectra so that they could be treated as signals corrupted by multiplicative Rayleigh-distributed white noise (Fig. 3i,j). Although the illumination angle for each full-field image is known, these values are not uniformly applicable to sub-regions unless the illumination wavefront is planar; hence, the illumination angle was estimated for each sub-region using a technique described in^36^. The sign of the illumination angle is ambiguous, but Eq. (3) is not sensitive to this sign change. Given $\widehat{\varphi _d}$, $\xi$ and the illumination angles, Eq. (3) can be rewritten as a function of only the local aberration coefficients. We retrieve these coefficients by solving an optimization problem using gradient descent from multiple random initializations (see “Methods”). This process is repeated for each sub-region of the FOV (see Fig. 4b), and the resulting field-varying aberration WEFs are shown in Fig. 4a.

The results from SHARP show that aberrations reach a minimum at the center of the FOV and increase progressively outward, as expected^37^. Averaged over the central 5-µm region, the total wavefront error was $0.0476\pm 0.0055$ waves rms (after the removal of residual defocus), corresponding to $\lambda /21 \, \hbox {rms}$ with a measurement accuracy within $\lambda$/182 (see “Methods” for error analysis). This result agrees with the nominal performance for a single-lens design, for which the region where the aberrations are contained below $\lambda$/20 is approximately $5\times 5 \upmu \, {\hbox {m}}^2$^38,39^. Defocus dominates along the vertical direction because of the off-axis geometry. Given the match to theoretical predictions for a single lens system, the aberration magnitude we found is expected to be dominated by the 5-µm measurement field size limit. In practice, such microscopes typically limit the quality imaging region to the center 1 to 3 µm. These results allow an analysis of the tool performance beyond the experimental demonstration of diffraction-limited imaging^40^, using a readily available photomask blank.

## Extension to illumination wavefront characterization

**Figure 5:**
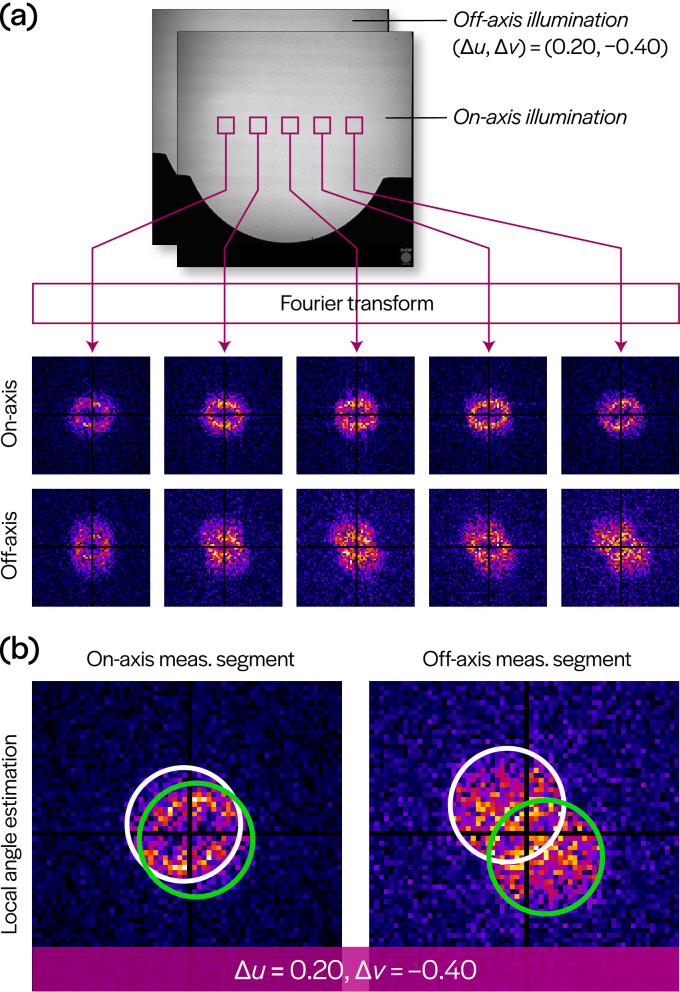
Field variation and disambiguation of local illumination angle. (**a**) Demonstration of the variation in computed Fourier spectrum magnitudes across the field in a single image, shown for both on- and off-axis illuminated images. (**b**) For a pair of images of the same sub-region, a known change in illumination angle $\left( \Delta u, \Delta v \right)$ disambiguates which circles correspond to the correct illumination angles (green) since only one pair of circles will have the correct relative shift.

In the aberration characterization procedure outlined above, illumination angles are estimated from data independently for each sub-region within the FOV. These angles need not be identical for different sub-regions of the same image, nor do they need to agree with the inputs to the illumination-steering hardware. Figure 5a demonstrates variation in local illumination angles across the FOV via changes in the positions of circular pupil support regions for different segments of the same full-field image.

Based on the preceding observation, we posit a method for reconstruction of the illumination wavefront from the local angle estimates in a manner similar to the analysis of Hartmann wavefront sensor data. Assuming that the illumination wavefront can be treated as locally planar, we can again segment the full-field image into smaller sub-regions. In this case, we reduce the sub-region size to $128 \times 128$ pixels to improve the localization of angle estimates, thereby increasing the resolution of the reconstructed wavefront. This sub-region size is too small for the aberration recovery procedure since interference patterns are not adequately sampled; however, it is sufficient for determining local illumination angle. To recover the angles, we estimate the domain containing the two instances of the pupil function in Eq. (3). While the sign ambiguity of the illumination angle does not affect aberration recovery, it is problematic in this case. Specifically, between the two circles identified by the angle estimation procedure, we need to determine which one corresponds to the angle with the correct sign. This can be resolved by using two images with a known relative change (see Fig. 5b), which is taken to be the difference in the input illumination angles of the acquired images. Of the four circles identified in the two images being considered, only one pair will be related by the known relative change; this removes the potential sign ambiguity in both images. Once the local illumination angle estimates are obtained, these values can be numerically integrated to retrieve a two-dimensional illumination wavefront.

An important caveat is that the procedure described above is only directly applicable to telecentric imaging systems. However, because SHARP is a non-telecentric single-lens imaging system, the local angle estimates contain a significant contribution from variation of the chief ray angle across the FOV. Note that in a telecentric system, the chief ray angle is always zero, so this effect can be ignored. Effectively accounting for this non-telecentricity is the subject of future work.

## Conclusion

We are able to reconstruct the field-dependent aberrations of a full-field EUV microscope using the atomic-scale roughness of photomask blanks and no additional hardware. Our results demonstrate that SHARP achieves diffraction-limited performance, with wavefront errors below $\lambda$/21 averaged over the center 5 µm $\times$ 5 µm region of the total captured field-of-view. We also demonstrated a measurement accuracy better than 4.0% ($\lambda$/181). This analysis was performed using only images acquired under standard operation of the microscope, and is useful when invasive techniques are difficult or impossible to implement, as is often the case for systems in ultra-high vacuum. This work demonstrates that our technique is suitable for evaluating the performance of the next generation of industrial-grade microscopes that will be used in semiconductor manufacturing. As X-ray light source facilities progress towards diffraction-limited storage rings and free electron lasers,with high brightness, this versatile, in-situ technique will prove increasingly valuable in the characterization of coherent sources and beamline optical systems.

## Methods

### SHARP EUV microscope

The SHARP EUV microscope objective is an off-axis zone plate with a focal length of 500 µm, that is manufactured at the Center for X-ray Optics. The zone plate achieves an NA of 0.082, and its $6^\circ$ off-axis geometry prevents the specular beam from reaching the sensor (see Fig. 1). The image of the sample is formed on a back-thinned CCD camera (PIXIS:2048, Princeton Instruments) located 450 mm downstream, providing an effective 900$\times$ magnification. The illumination angle-scanning mirror is 1 mm x 1 mm MEMS device (Mirrorcle Technologies) coated with an Mo/Si reflective multilayer tuned for the $55^\circ$ nominal angle of operation. A elliptical condenser mirror is placed such that the angle-scanning mirror is conjugate to the object plane. Angle scanning during image acquisition is used to improve the uniformity of the illumination and reduce coherent artifacts. The microscope operates on a bend magnet at Beamline 11.3.2 of the ALS, at a wavelength of 13.5 nm (91.7 eV) with a bandwidth of 1:1450, under ultra-high vacuum conditions.

### Gradient descent for aberration recovery

The calibration (defocused) measurement is taken such that defocus is the dominant term in the aberration function. This can be verified by the existence of its characteristic concentric rings in the Fourier spectrum^24^, which can be seen in Fig. 2b (image labeled ‘defocused’). By fitting a low-dimensional model to the spectrum^25^, we can estimate the deterministic Gaussian support $|\widehat{\varphi _{d}}|$ and Rayleigh parameter $\xi$. We can then whiten all of the Fourier spectra to remove the effect of the Gaussian support. These whitened measurements are described by:

4 $$\begin{aligned} {\mathbf {m}}_{j} = \dfrac{|\widehat{I_{\varnothing ,j}}|}{2 \cdot | \widehat{\varphi _{d}} |}. \end{aligned}$$

To recover aberrations for a particular sub-region, in the form of a ($\pi$-normalized, OSA/ANSI ordered) Zernike coefficient vector, ${\mathbf {c}}$, we formulate a nonlinear least squares (NLS) inverse problem based on Eqs. (3) and (4), which is derived in^25^. The problem can be written as:

5 $$\begin{aligned} {\mathbf {c}}^{\star } = {\text {arg}} \min _{{\mathbf {c}}} ~ \sum \limits _{j=1}^{K} \left\Vert ~ {\mathbb {1}}[{\mathscr {U}}_{j}] \circ \left( {\mathbf {m}}_{j} - {\mathbb {E}} \left[ \eta \right] \Big | \sin \big ( {\mathbf {A}}_{j} {\mathbf {c}} \big ) \Big | \right) \right\Vert ^2, \end{aligned}$$

where ${\mathbf {A}}_{j}$ maps aberration coefficients to a sampled self-interference pattern, ${\mathscr {U}}_{j}$ is a set which describes the support containing the interference pattern (overlap of two circles), ${\mathbb {1}}[\cdot ]$ denotes a characteristic (indicator) function for a set, ${\mathbb {E}}[\cdot ]$ denotes an expectation and *K* is the number of measurements used. The structure of ${\mathscr {U}}_{j}$ and ${\mathbf {A}}_{j}$ is determined by the (known) illumination angles indexed by *j*. Plots of the functions $\frac{{\mathbb {1}}[{\mathscr {U}}_{j}]}{2\cdot | \widehat{\varphi _{d}} |}$ are shown in Fig. 3g,h. Plots of the functions ${\mathbb {1}}[{\mathscr {U}}_{j}] \cdot {\mathbf {m}}_j$ are shown in Fig. 3i,j; note the existence of a zero-frequency component which is digitally removed from consideration by our algorithm. To guarantee uniqueness of the recovered aberration polynomial, we need $K \ge 3$; in our experiments, we use $K = 9$. Using more measurements generally improves the robustness of the approach but can be replaced with more initializations.

**Figure 6:**
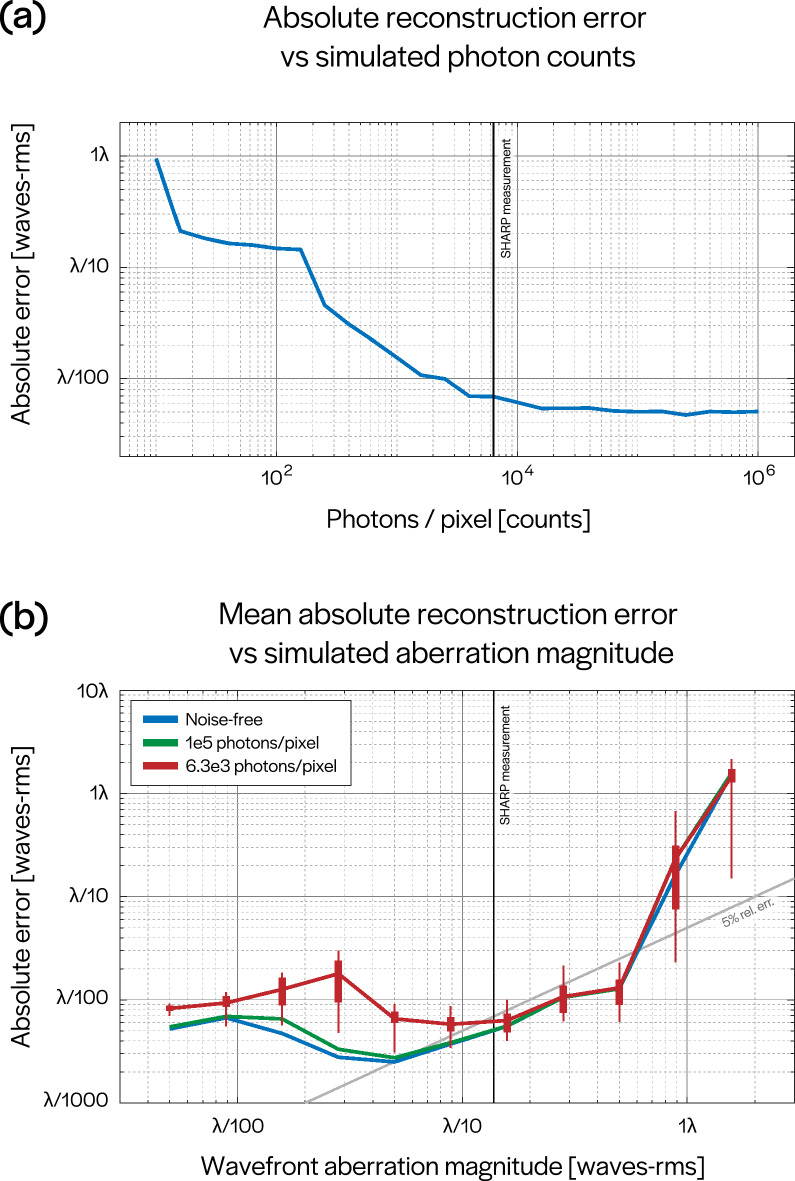
Reconstruction error analysis. (**a**) Relative reconstruction error for a single simulated WEF (magnitude 0.158 waves rms) and images corrupted by varying levels of shot noise. The vertical black line represents the imaging conditions of SHARP, roughly 6,300 photons/pixel. (**b**) Mean absolute reconstruction error for 25 independent WEFs at each of several magnitudes of rms wavefront error. The analysis was performed without adding noise to images (blue), simulating experimental conditions of 6,300 photons/pixel (red) and simulating $10^5$ photons/pixel—corresponding roughly to averaging 16 images at each illumination angle. The minima, maxima and interquartile ranges of absolute errors for simulations at 6,300 photons/pixel are also shown.

We solve the optimization problem formulated above by using gradient descent with multiple random initializations. For each image sub-region, we generate 125 random vectors with Gaussian-distributed elements as initialization points. From each, we compute 200 iterations of gradient descent using backtracking line search^41^ and select the vector with minimum cost, given by the right-hand-side of Eq. (5).

### Error analysis in aberration characterization

To estimate the reconstruction error in our aberration recovery algorithm, we simulated a set of measurements based on the sub-region size we consider ($256 \times 256$) and the parameters of the SHARP imaging system (NA, wavelength, magnification and illumination angles). To characterize the effects of shot noise, we simulated measurements of a fixed aberration polynomial with various levels of photon counts per pixel and attempted to recover the WEF. For each level, we initialized the algorithm with 50 randomly chosen points, selected the converged result with minimum cost and recorded its error. The aberration magnitude used roughly corresponds to the value measured in the sweet spot of SHARP (roughly 0.158 waves-rms, or $\approx \lambda /6 \, \hbox {rms}$, including defocus). The results of these trials are shown in Fig. 6a, in which the vertical black line corresponds to the imaging conditions of SHARP—roughly 6,300 photons/pixel. At this level, the absolute reconstruction error ($\varepsilon _a = \frac{1}{2}||{\mathbf {c}} - \mathbf {c^*}||_2$) of the reported coefficient vector was $0.0069\lambda$ waves rms ($\lambda /145 \, \hbox {rms}$), corresponding to a relative error ($\varepsilon _r = ||{\mathbf {c}} - \mathbf {c^*}||_2/||{\mathbf {c}}||_2$) of 4.3%.

To characterize the performance of our algorithm at various magnitudes of system aberrations, we generated 25 datasets at each of 11 levels of rms wavefront error. For each dataset, we initialize our algorithm with 50 random vectors with approximately the same magnitude as the true coefficient vector, and we report the converged solution with minimum cost (see Eq. 5) as the recovered Zernike coefficient vector. We then note the absolute reconstruction errors for each of the 25 reported solutions. We performed an identical analysis under three different levels of simulated shot noise: noise-free, $10^5$ photons/pixel and experimental conditions (6,300 photons/pixel). The mean absolute reconstruction errors are shown in Fig. 6b, along with the minima, maxima and interquartile ranges for simulations under experimental illumination conditions. The vertical black line corresponds to the experimentally obtained aberration magnitude of SHARP (including defocus). At the nearest sampled aberration magnitude to this level (roughly 0.158 waves rms, approximately $\lambda /6 \, \hbox {rms}$), the simulated aberration polynomials were reconstructed with a mean absolute error of 0.0063 waves-rms ($\lambda /159 \, \hbox {rms}$), corresponding to a mean relative error of 4.0%. As a result, we claim that the true aberrations in the sweet spot of SHARP lie within 4.0% of our reconstruction.

In the sweet spot of SHARP, we recover a local aberration WEF magnitude of 0.138 waves rms (approximately $\lambda /7 \, \hbox {rms}$), which is mostly due to a defocus coefficient of 0.130 waves rms. Computing a 4.0% relative error, we have an uncertainty of 0.0055 waves rms ($\lambda /182 \, \hbox {rms}$), which we report in the main text.

## Data Availability

The data and the reconstruction procedure presented in this paper are available at github.com/gautamgunjala.
